# Results from the 32‐week, phase 3 DISCREET study of apremilast in patients with moderate to severe genital psoriasis

**DOI:** 10.1111/jdv.70110

**Published:** 2025-12-10

**Authors:** Joseph F. Merola, Lyn Guenther, Charles Lynde, Kim A. Papp, Lawrence Charles Parish, Paul Yamauchi, Sue Cheng, Hamid Amouzadeh, Cynthia Deignan, Shauna Jardon, Mindy Chen, Andreas Pinter

**Affiliations:** ^1^ Division of Rheumatology, Department of Dermatology UT Southwestern Medical Center Dallas Texas USA; ^2^ Division of Rheumatology, Department of Medicine UT Southwestern Medical Center Dallas Texas USA; ^3^ Guenther Research Inc. London Ontario Canada; ^4^ Lynde Institute for Dermatology Markham Ontario Canada; ^5^ Probity Medical Research Markham Ontario Canada; ^6^ Probity Medical Research Waterloo Ontario Canada; ^7^ Alliance Clinical Trials Waterloo Ontario Canada; ^8^ Division of Dermatology, Temerty Faculty of Medicine University of Toronto Toronto Ontario Canada; ^9^ Paddington Testing Company, Inc. Philadelphia Pennsylvania USA; ^10^ Dermatology Institute & Skin Care Center Santa Monica California USA; ^11^ Amgen Inc. Thousand Oaks California USA; ^12^ University Hospital of the Goethe University Frankfurt am Main Germany

**Keywords:** apremilast, clinical trial, genitalia, psoriasis, randomized controlled trial

## Abstract

**Background:**

Genital psoriasis is highly prevalent among patients with psoriasis, is often stigmatized, causes pain and discomfort and negatively impacts quality of life. Apremilast is an oral phosphodiesterase 4 inhibitor approved for treating psoriasis and has demonstrated safety and efficacy in treating genital psoriasis, as seen in the primary 16‐week DISCREET results.

**Objectives:**

To assess the efficacy and safety of apremilast 30 mg twice daily in patients with moderate to severe genital psoriasis over the 32‐week study duration.

**Methods:**

DISCREET was a phase 3, multicentre, randomized, double‐blind trial that evaluated apremilast 30 mg twice daily versus placebo in a 16‐week placebo‐controlled phase (randomization 1:1) followed by a 16‐week apremilast extension phase. Patients had moderate to severe genital psoriasis, defined as a modified static Physician's Global Assessment of Genitalia (genital PGA) score of ≥3. They also either had disease inadequately controlled by or were intolerant to topical therapy. We report the results through Week 32.

**Results:**

Of 289 patients randomized, 229 continued to the apremilast extension phase (Weeks 16–32): 110 in the placebo/apremilast group and 119 in the apremilast/apremilast group. At 32 weeks, 51.8% (95% CI: 42.6, 60.9) of patients in the placebo/apremilast group and 40.3% (95% CI: 32.0, 49.3) of patients in the apremilast/apremilast group had achieved a modified genital PGA response (score of 0/1 with ≥2‐point reduction from baseline). At Week 32, similar improvements in skin, genital signs and symptoms and quality of life were observed in patients who started apremilast at Week 16 or at randomization. Frequently reported treatment‐emergent adverse events during all‐apremilast exposure were diarrhoea (25.4%), nausea (19.4%) and headache (17.9%).

**Conclusions:**

Apremilast is an effective oral systemic therapy in patients with moderate to severe genital psoriasis and has shown consistent clinical efficacy, lessening of symptoms and quality‐of‐life benefit in DISCREET.

**Clinical trial **ID**:**

NCT03777436.

## Plain Language Summary

Patients with psoriasis often have symptoms in the genital area. Genital psoriasis can cause uncomfortable symptoms such as itching, burning, flaking, and pain, as well as embarrassment and negative effects on sexual and mental health. Few clinical studies have focused on treatments specifically for genital psoriasis. Steroid creams are commonly prescribed, however some patients dislike this treatment due to side effects, inconvenience, messiness, and lack of effectiveness.

In the multinational DISCREET study, we examined how effective and safe ‘apremilast’ (an oral medication) was in treating moderate to severe genital psoriasis. We previously reported that apremilast was safe and effective over 16 weeks compared to ‘placebo’ (a dummy drug containing no active ingredients). Here we report the long‐term results over 32 weeks. In total, 289 patients received either apremilast or placebo for 16 weeks, after which all patients received apremilast until week 32.

At 32 weeks, nearly half (46%) of all patients achieved clear or almost clear genital skin; patients who received apremilast throughout the entire 32‐week study and patients who switched from placebo to apremilast at 16 weeks experienced similar effectiveness. Patients also had improvements in genital itch and other symptoms, as well as in general and sexual well‐being. Both women and men benefited from apremilast, with the benefit more pronounced in women. No new safety concerns were identified compared to previous studies.

In conclusion, these findings indicate that apremilast is an effective and safe oral medication for patients with moderate to severe genital psoriasis.

## Graphical abstract

During the extension phase of the 32‐week DISCREET trial, apremilast treatment in patients with moderate to severe genital psoriasis led to clinically meaningful improvements in disease severity (modified genital PGA, overall sPGA, BSA), symptoms (genital itch, GPSS), and quality of life (DLQI, DLQI‐Q9), with safety outcomes consistent with prior studies.

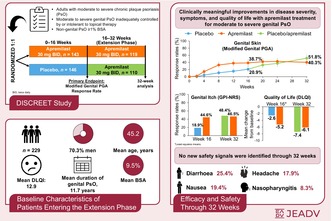

**Why was the study undertaken?**

DISCREET was designed to determine the safety and efficacy of apremilast for treating patients with moderate to severe genital psoriasis.

**What does this study add?**

Apremilast treatment led to improvements in clinical findings (genital PGA, sPGA, BSA), symptoms [GPI‐NRS (genital itch), GPSS (genital psoriasis symptoms)] and quality of life (DLQI, DLQI‐Q9) in patients with genital psoriasis over 32 weeks. Safety outcomes were in line with the known safety profile of apremilast.

**What are the implications of this study for disease understanding and/or clinical care?**

Genital psoriasis, a highly burdensome disease, significantly impacts quality of life. Apremilast, an oral phosphodiesterase 4 inhibitor, is an effective treatment option for patients with moderate to severe genital psoriasis.

## INTRODUCTION

Genital psoriasis can affect over half of individuals with psoriasis during the course of their disease^1^, ^2^ and is often highly stigmatized.^3^ Patients often experience bothersome physical signs and symptoms such as itching, burning, cracking, scaliness, redness, induration and pain, as well as negatively impacted sexual health, mental health and health‐related quality of life (QoL).^1^, ^4^, ^5^ Nearly half of respondents to one survey reported that they have not discussed their genital psoriasis with their physician, and more than two thirds reported that they have never had treatment for genital lesions.^4^

Limited data support treatments specifically for genital psoriasis. Topical therapies, such as corticosteroids, are typically used first‐line; however, compliance with topical applications may not be ideal due to the risk of adverse effects on sensitive genital skin, the burden of application and/or poor efficacy.^6^, ^7^

Apremilast is an oral immunomodulating phosphodiesterase 4 inhibitor approved for treating chronic plaque psoriasis.^8^, ^9^ DISCREET was the first randomized, double‐blind, placebo‐controlled trial evaluating an oral systemic therapy in patients with genital psoriasis using clinical measures of genital psoriasis. The primary 16‐week DISCREET results have previously demonstrated the safety and efficacy of apremilast treatment.^10^ Product labelling in the United States has been revised to include these data supporting the efficacy of apremilast in genital psoriasis. We report 32‐week safety and efficacy results as well as post‐hoc analyses of efficacy results.

## MATERIALS AND METHODS

### Study design

DISCREET (NCT03777436) was a phase 3, multicentre, randomized, placebo‐controlled, double‐blind trial conducted in Belgium, Canada, France, Germany, Italy and the United States. Methods have previously been reported.^10^ Briefly, patients were randomized 1:1 to apremilast or placebo based on a permuted block randomization using centralized Interactive Response Technology. Patients received oral apremilast 30 mg or matched placebo twice daily in a 16‐week placebo‐controlled phase, followed by a 16‐week extension phase during which all patients continued (referred to as the apremilast/apremilast group [treated with apremilast from Weeks 0–32]) or transitioned to apremilast (referred to as the placebo/apremilast group [received apremilast from Weeks 16–32]). Visits were scheduled at screening, baseline, Weeks 2, 4, 8, 12, 16, 20, 24 and 32 (±4 days, each) and 4 weeks following the last treatment dose (±2 weeks).

This study was approved by an institutional review board or independent ethics committee before commencement and conducted in compliance with Good Clinical Practice, the International Council for Harmonization Guideline E6, the Declaration of Helsinki and any applicable regulatory requirements. Before any study‐related procedures, patients provided signed informed consent.

### Eligibility criteria

DISCREET enrolled adults with moderate to severe genital psoriasis (defined as a baseline modified static Physician's Global Assessment of Genitalia [sPGA‐G, or genital PGA] score of ≥3)^11^ inadequately controlled by or intolerant to topical therapy, moderate to severe chronic plaque psoriasis (overall static Physician's Global Assessment [sPGA] score of ≥3) for ≥6 months from diagnosis and non‐genital plaque psoriasis with body surface area (BSA) ≥1%. Concomitant treatment for psoriasis, including topical therapy, conventional systemic therapy, phototherapy or biologic therapy, was not permitted for the duration of the study.

### Efficacy analyses

The primary efficacy endpoint was the proportion of patients achieving a modified genital PGA response (score of 0 [clear] or 1 [almost clear] with ≥2‐point reduction from baseline) at Week 16 (results previously reported^10^). Physician‐reported efficacy endpoints included the proportion of patients achieving an overall sPGA response (score of 0 or 1 [clear or almost clear] with ≥2‐point reduction from baseline) and the change from baseline in affected BSA. Patient‐reported efficacy endpoints included the proportion of patients with a ≥4‐point improvement in Genital Psoriasis Itch Numeric Rating Scale (GPI‐NRS) in patients with a baseline score ≥4 within the Genital Psoriasis Symptoms Scale (GPSS; scored 0 [no itch] to 10 [worst itch imaginable]), the change from baseline in GPSS total score (evaluates itching, pain, discomfort, stinging, burning, redness, scaling and cracking; scored 0 [no genital psoriasis symptoms] to 80 [worst genital psoriasis symptoms imaginable])^12^ and the change from baseline in Dermatology Life Quality Index (DLQI; scored 0–30, with higher scores indicating a more greatly impacted QoL)^13^, ^14^ and DLQI question 9 (DLQI‐Q9; ‘Over the last week how much has your skin caused any sexual difficulties?’; scored 0–3). Week 16 efficacy outcomes have been previously reported.^10^ Efficacy outcomes over 32 weeks are presented. Post‐hoc analyses, including efficacy outcomes by sex and agreement between clinical and QoL improvements with apremilast treatment, are described in the Supplemental Methods.

### Safety analysis

Safety was assessed throughout the study, from the time of signed informed consent to 28 days after the last dose of study treatment; the type, frequency and severity of adverse events were reported, and the relationship of the adverse event to study treatment was determined.

### Statistical analysis

Information on the determination of sample size and other details on statistical analyses of DISCREET are presented in the primary publication.^10^ Efficacy analyses for response rates (i.e. modified genital PGA, sPGA, GPI‐NRS) are based on the intent‐to‐treat population who entered the apremilast extension phase. Missing data were handled using non‐responder imputation. Change from baseline values (i.e. BSA, GPSS, DLQI, DLQI‐Q9) in the placebo‐controlled phase (Weeks 0–16) are presented as least‐squares means and based on the intent‐to‐treat population and mixed effects model for repeated measures; values during the apremilast extension phase (Weeks 16–32) are presented as means based on data as observed. For subgroup analyses by sex, Week 16 data are presented for the intent‐to‐treat population and missing data were imputed using multiple imputations; data at Week 32 are given as observed. The subgroups for binary endpoints at Week 16 were analyzed using the Cochran–Mantel–Haenszel (CMH) test adjusted for the stratification factor at baseline (BSA <10% or ≥10%). Treatment differences at Week 16 were based on the weighted average of the treatment differences across the strata with the CMH weights, and the associated two‐sided 95% CIs were based on the normal approximation to the weighted average.

Safety outcomes are based on apremilast patients as treated, including any patient who was treated with ≥1 dose of apremilast in either the placebo‐controlled or apremilast extension phase. Exposure‐adjusted incidence rates per 100 patient‐years are presented and defined as 100 times the number of patients reporting the event divided by patient‐years within the phase, up to the first event start date for patients reporting the event. Adverse events were coded using MedDRA version 24.1.

## RESULTS

### Patient population

**FIGURE 1:**
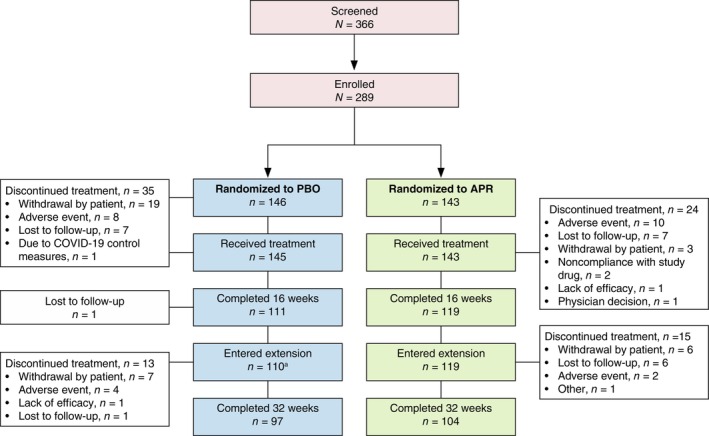
CONSORT diagram of the DISCREET trial. APR, apremilast 30 mg twice daily; PBO, placebo. ^a^A total of 109 patients received ≥1 dose of apremilast.

Between February 2019 and February 2022, 289 patients across 49 sites were enrolled in the DISCREET trial, including 146 patients randomized to placebo and 143 to apremilast (Figure 1). Baseline characteristics for the intent‐to‐treat population have previously been reported.^10^ A total of 229 patients (79.2%) entered the apremilast extension phase (placebo/apremilast, *n* = 110 [75.3%]; apremilast/apremilast, *n* = 119 [83.2%]), with 201 (69.6%) completing 32 weeks of treatment (placebo/apremilast, *n* = 97 [66.4%]; apremilast/apremilast, *n* = 104 [72.7%]). Baseline characteristics for the patients entering the apremilast extension phase are summarized in Table 1. The proportion of men was 2.4 times that of women. Mean baseline DLQI (12.9) indicated a population with a QoL substantially impacted by their disease.^14^

**TABLE 1:** Baseline demographics and clinical characteristics of patients who entered the apremilast extension phase.

	Placebo/apremilast *n* = 110	Apremilast/apremilast *n* = 119	Total *N* = 229
*Age, years*			
Mean (SD)	47.2 (14.3)	43.4 (13.6)	45.2 (14.0)
Median (IQR)	47.5 (36, 57)	42.0 (33, 55)	44.0 (34, 56)
*Sex, n (%)*			
Male	76 (69.1)	85 (71.4)	161 (70.3)
Female	34 (30.9)	34 (28.6)	68 (29.7)
*Weight, kg*			
Mean (SD)	87.4 (20.3)	92.0 (21.4)	89.8 (20.9)
Median (IQR)	85.9 (74.8, 97.2)	88.7 (80.7, 102.0)	88.1 (77.1, 100.2)
*Body mass index, kg/m* ^*2*^			
Mean (SD)	29.7 (5.8)	30.4 (6.7)	30.1 (6.3)
Median (IQR)	28.8 (25.4, 33.9)	29.2 (26.3, 33.1)	29.0 (25.9, 33.4)
*Duration of psoriasis, years*			
Mean (SD)	15.4 (13.7)	15.0 (12.5)	15.2 (13.1)
Median (IQR)	11.1 (3.9, 23.5)	10.7 (4.1, 21.7)	11.0 (4.0, 22.2)
*Duration of genital psoriasis, years*			
Mean (SD)	12.5 (13.3)	11.0 (11.5)	11.7 (12.4)
Median (IQR)	7.3 (2.7, 18.3)	6.5 (2.6, 18.3)	6.8 (2.6, 18.3)
*Modified genital PGA score, n (%)*			
3 (moderate)	96 (87.3)	101 (84.9)	197 (86.0)
4 (severe)	14 (12.7)	18 (15.1)	32 (14.0)
*Overall sPGA score, n (%)*			
3 (moderate)	98 (89.1)	105 (88.2)	203 (88.6)
4 (severe)	11 (10.1)	14 (11.8)	25 (10.9)
*BSA, %*			
Mean (SD)	8.3 (4.9)	10.7 (13.2)	9.5 (10.1)
Median (IQR)	8.0 (4.0, 11.0)	7.0 (5.0, 12.0)	7.0 (4.0, 12.0)
<10%, *n* (%)	64 (58.2)	68 (57.1)	132 (57.6)
≥10%, *n* (%)	46 (41.8)	51 (42.9)	97 (42.4)
*DLQI*			
Mean (SD)	12.8 (6.5)	12.9 (6.7)	12.9 (6.6)
Median (IQR)	11.0 (8.0, 18.0)	12.0 (8.0, 17.0)	12.0 (8.0, 17.0)
*DLQI‐Q9*			
Mean (SD)	1.4 (1.1)	1.4 (1.1)	1.4 (1.1)
Median (IQR)	1.0 (0.0, 2.0)	1.0 (0.0, 2.0)	1.0 (0.0, 2.0)
*GPI‐NRS*			
Mean (SD)	6.4 (2.5)	6.6 (2.3)	6.5 (2.4)
Median (IQR)	7.0 (5.0, 8.0)	7.0 (5.0, 8.0)	7.0 (5.0, 8.0)

*Note*: The *n* values reflect the number of patients who entered the apremilast extension phase; the actual number of patients available for each parameter may vary.

Abbreviations: BSA, body surface area; DLQI, Dermatology Life Quality Index; Genital PGA, static Physician's Global Assessment of Genitalia; GPI‐NRS, Genital Psoriasis Itch Numeric Rating Scale; IQR, interquartile; Q9, question 9; SD, standard deviation; sPGA, static Physician's Global Assessment.

### Efficacy outcomes

#### Physician‐reported

**FIGURE 2:**
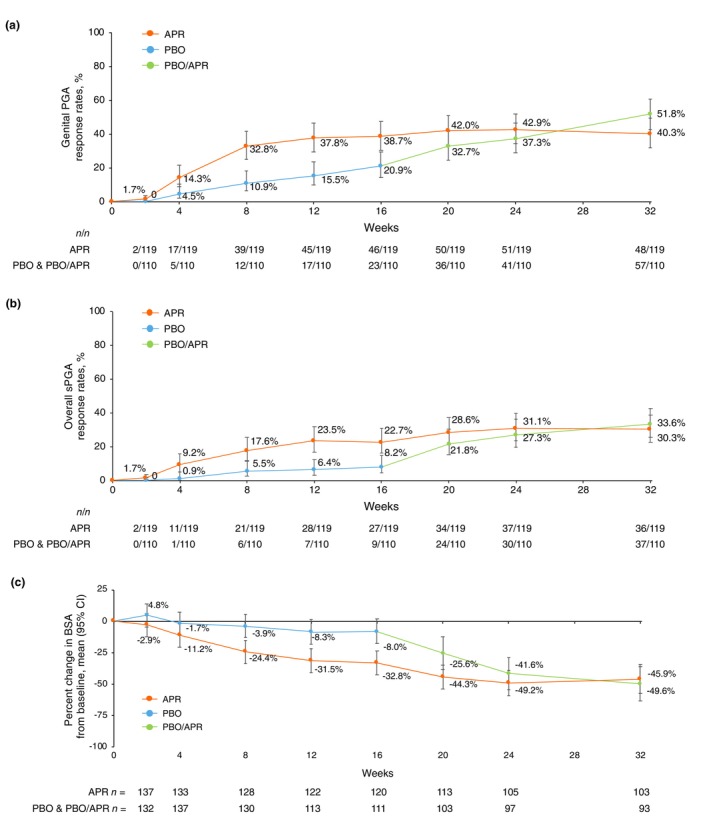
Physician‐reported outcomes over 32 weeks, including modified genital PGA responses (score of 0/1 with ≥2‐point reduction from baseline) (a), overall sPGA responses (score of 0/1 with ≥2‐point reduction from baseline) (b), and percent change from baseline in BSA (c). Data shown in panels (a, b) are based on patients who entered the apremilast extension phase with non‐responder imputation for missing data. Data shown in panel (c) are least‐squares means based on the mixed effects model for repeated measures during the PBO‐controlled period (Weeks 0–16) and based on data as observed during the apremilast extension phase (Weeks 16–32). Error bars represent 95% CI. APR, apremilast 30 mg twice daily; BSA, body surface area; CI, confidence interval; genital PGA, static Physician's Global Assessment of Genitalia; PBO, placebo; sPGA, static Physician's Global Assessment.

Efficacy outcomes for the 16‐week placebo‐controlled phase have been previously reported.^10^ Among those in the apremilast extension phase, modified genital PGA and overall sPGA response rates were greater at Week 16 for patients randomized to apremilast compared with placebo and were maintained or improved between Weeks 16 and 32 in the apremilast extension phase. At Week 32, 51.8% (*n*/*N* = 57/110; 95% CI: 42.6, 60.9) of patients in the placebo/apremilast group and 40.3% (*n*/*N* = 48/119; 95% CI: 32.0, 49.3) in the apremilast/apremilast group achieved a modified genital PGA response (Figure 2a). Overall sPGA response was achieved in 33.6% (*n*/*N* = 37/110; 95% CI: 25.5, 42.9) of patients in the placebo/apremilast group and 30.3% (*n*/*N* = 36/119; 95% CI: 22.7, 39.0) in the apremilast/apremilast group at Week 32 (Figure 2b). Percent change in BSA over time is shown in Figure 2c and demonstrates a sustained decrease in BSA between Weeks 16 and 32 in both groups. Mean change in BSA from baseline at Week 32 was −4.4 (95% CI: −5.6, −3.3) for the placebo/apremilast group (*n* = 93; baseline mean: 8.3%) and −5.3 (95% CI: −7.0, −3.6) for the apremilast/apremilast group (*n* = 103; baseline mean: 10.7%). Post‐hoc analyses demonstrated that 29.9% of patients overall (31.9% in the placebo/apremilast group, 28.2% in the apremilast/apremilast group) achieved full genital skin clearance at Week 32 (Figure S1A).

#### Patient‐reported

**FIGURE 3:**
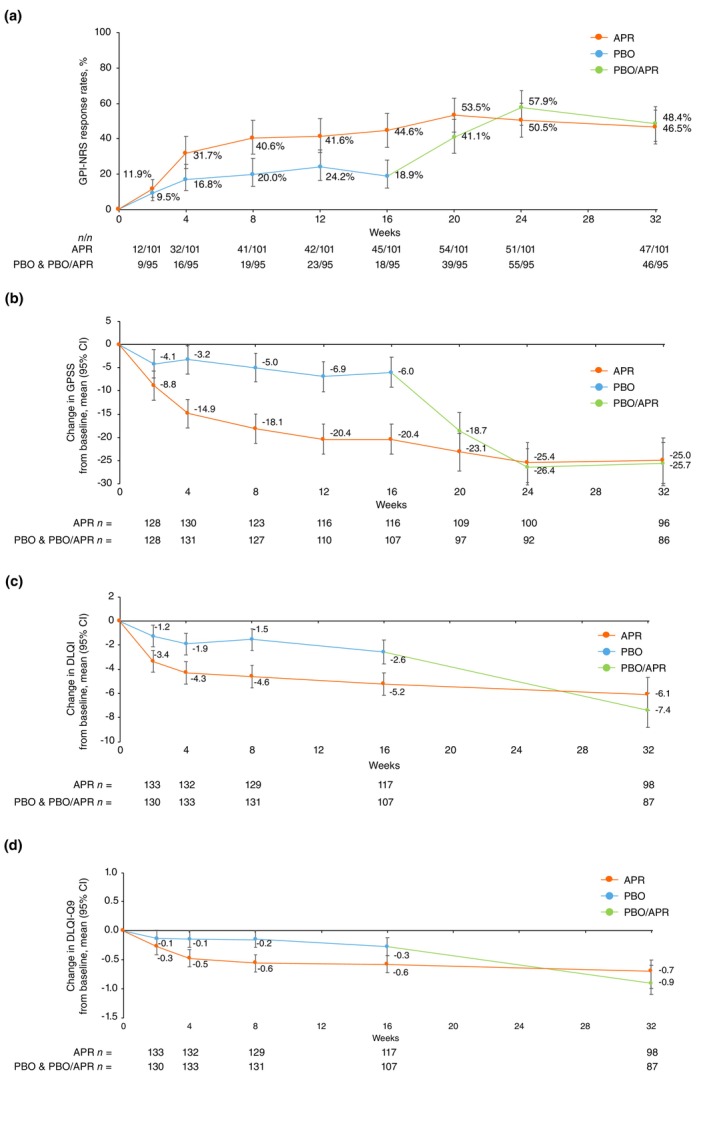
Patient‐reported outcomes over 32 weeks, including GPI‐NRS responses (a), change from baseline in GPSS total score (b), change from baseline in DLQI (c) and change from baseline in DLQI‐Q9 (d). Data shown in panel (a) are based on patients who entered the apremilast extension phase with non‐responder imputation for missing data. Panel (a) includes patients with a baseline GPI‐NRS score of ≥4. Data shown in panels (b–d) are least‐squares means based on the mixed effects model for repeated measures during the PBO‐controlled phase (Weeks 0–16) and based on data as observed during the apremilast extension phase (Weeks 16–32). Error bars represent 95% CI. APR, apremilast 30 mg twice daily; CI, confidence interval; DLQI, Dermatology Life Quality Index; DLQI‐Q9, Dermatology Life Quality Index Question 9; GPI‐NRS, Genital Psoriasis Itch Numeric Rating Scale; GPSS, Genital Psoriasis Symptoms Scale; PBO, placebo.

Response rates of GPI‐NRS and mean change from baseline in GPSS, DLQI and DLQI‐Q9 were greater at Week 16 with apremilast versus placebo in the placebo‐controlled phase and were maintained or improved during the extension phase. Among patients with a baseline GPI‐NRS ≥4, 48.4% (*n*/*N* = 46/95; 95% CI: 38.6, 58.3) and 46.5% (*n*/*N* = 47/101; 95% CI: 37.1, 56.2) of patients in the placebo/apremilast and apremilast/apremilast groups, respectively, achieved GPI‐NRS response at Week 32 (Figure 3a). The mean change from baseline in GPSS total score at Week 32 was −25.7 (95% CI: −30.4, −21.1) in the placebo/apremilast group (*n* = 86) and −25.0 (95% CI: −29.9, −20.1) in the apremilast/apremilast group (*n* = 96) (Figure 3b). At Week 32, mean change from baseline in DLQI was −7.4 (95% CI: −8.8, −6.0) in the placebo/apremilast group (*n* = 87) and −6.1 (95% CI: −7.4, −4.7) in the apremilast/apremilast group (*n* = 98) (Figure 3c); mean change in DLQI‐Q9 was −0.9 (95% CI: −1.1, −0.6) and −0.7 (95% CI: −1.0, −0.5), respectively (Figure 3d). In post‐hoc analyses, 10.1% of patients treated with placebo (*n*/*N* = 11/109) and 18.3% treated with apremilast (*n*/*N* = 22/120) achieved a DLQI score of 0 or 1 (range 0–30) at Week 16; at Week 32, 27.4% of patients overall (*n*/*N* = 52/190; 29.2% in the placebo/apremilast group, 25.7% in the apremilast/apremilast group) achieved a DLQI score of 0 or 1 (Figure S1B). When excluding patients with a baseline DLQI‐Q9 score of 0 (range 0–3), 25.0% (*n*/*N* = 20/80) of patients treated with placebo and 37.8% (*n*/*N* = 31/82) of patients treated with apremilast achieved a DLQI‐Q9 score of 0 at Week 16. At Week 32, 50.4% of patients overall (*n*/*N* = 67/133; 47.7% placebo/apremilast, 52.9% apremilast/apremilast) achieved a DLQI‐Q9 score of 0 (Figure S1C).

#### Outcomes by sex

At randomization, 102 men and 44 women were assigned to placebo and 100 men and 43 women to apremilast (Table S1). Both men and women treated with apremilast during the placebo‐controlled phase had higher response rates at Week 16 than those in the placebo group. Treatment differences were 17.1% (95% CI: 4.3, 29.8) in males and 27.3% (95% CI: 6.7, 47.8) in females for modified genital PGA response, 16.1% (95% CI: 7.0, 25.2) in males and 13.4% (95% CI: −4.3, 31.0) in females for overall sPGA response, and 21.7% (95% CI: 6.7, 36.8) in males and 40.0% (95% CI: 21.7, 58.2) in females for GPI‐NRS response. At 32 weeks, 44.7% of males and 48.5% of females had a modified genital PGA response (Figure S2A), 28.6% of men and 39.7% of women had an overall sPGA response (Figure S2B), and 42.0% of men and 58.5% of women had a GPI‐NRS response (Figure S2C).

Both men and women had greater improvement in DLQI and DLQI‐Q9 scores after 16 weeks of apremilast compared with placebo. Treatment differences were −2.6 (95% CI: −4.5, −0.8) in men and −2.8 (95% CI: −5.7, 0.1) in women for change from baseline in total DLQI and −0.3 (95% CI: −0.5, −0.0) in men and −0.4 (95% CI: −0.8, 0.0) in women for change from baseline in DLQI‐Q9. At 32 weeks, mean change from baseline in total DLQI score was −5.6 (95% CI: −6.7, −4.4) in men and −9.3 (95% CI: −11.1, −7.5) in women (Figure S2D) and mean change from baseline in DLQI‐Q9 score was −0.7 (95% CI: −0.9, −0.5) in men and −1.1 (95% CI: −1.4, −0.8) in women (Figure S2E).

#### Agreement between clinical and QoL improvements with apremilast

Agreement between improvements in clinical outcomes and symptoms (modified genital PGA score of 0, overall sPGA score of 0, or GPI‐NRS response) and improvements in QoL outcomes (DLQI or DLQI‐Q9 scores of 0 or 1) with apremilast treatment at Weeks 16 and 32 was determined (Table S2). Among all combinations of clinical and QoL improvements assessed, QoL outcomes had the highest agreement with improvement in GPI‐NRS response. At Week 32, 20.2% of patients with available data for both endpoints achieved both a DLQI score of 0 or 1 and a GPI‐NRS response, and 52.4% achieved both a DLQI‐Q9 score of 0 or 1 and a GPI‐NRS response.

### Safety outcomes

Safety outcomes for the 16‐week placebo‐controlled phase have been previously reported.^10^ Safety analyses were performed for patients treated with ≥1 dose of apremilast at any time during the 32‐week DISCREET study: 252 patients received apremilast at any time, for a total of 107.0 patient‐years of exposure. The incidence of treatment‐emergent adverse events (TEAEs) throughout apremilast exposure remained similar to that in the placebo‐controlled phase. The most frequently reported TEAEs (in ≥5% of patients) were diarrhoea (25.4%), nausea (19.4%), headache (17.9%) and nasopharyngitis (8.3%), which similarly aligned with the placebo‐controlled phase (Table 2).^10^ Serious TEAEs were reported in a total of 5 patients between Weeks 1 and 8 (*n* = 2), Weeks 8 and 16 (*n* = 2), and Weeks 24–32 (*n* = 1). TEAEs that led to treatment withdrawal in more than 1 patient were nausea (*n* = 6), diarrhoea (*n* = 4), depression (*n* = 3) and decreased appetite (*n* = 2).

**TABLE 2:** Safety overview of patients treated with apremilast at any time on study.

	Apremilast patients as treated *N* = 252, PY = 107.0	
	*n* (%)	EAIR/100 PY
Any TEAE	174 (69.0)	424.5
Any serious TEAE	5 (2.0)	4.7
Any serious treatment‐related TEAE	1 (0.4)	0.9
Any TEAE leading to treatment withdrawal	16 (6.3)	15.0
TEAEs occurring in ≥5% of patients		
Diarrhoea	64 (25.4)	77.5
Nausea	49 (19.4)	56.3
Headache	45 (17.9)	50.6
Nasopharyngitis	21 (8.3)	20.9

*Note*: EAIR per 100 PY is defined as 100 times the number of patients reporting the specific event divided by PY within the phase (up to the first event start date for patients reporting the event).

Abbreviations: EAIR, exposure‐adjusted incidence rate; PY, patient‐years; TEAE, treatment‐emergent adverse event.

## DISCUSSION

Patients with moderate to severe genital psoriasis treated with apremilast in the phase 3 DISCREET study had meaningful improvements in disease symptoms and severity, and QoL. DISCREET is the first randomized, double‐blind, placebo‐controlled clinical trial designed and powered to study an oral systemic therapy in patients with genital psoriasis using genital‐specific clinical measures (e.g. genital PGA, GPSS, GPI‐NRS). Primary results showed that 39.6% of patients treated with apremilast versus 19.5% of patients treated with placebo achieved the primary endpoint of modified genital PGA response (score of 0 or 1 with ≥2‐point reduction from baseline) at Week 16.^10^ Patients treated with placebo during the first 16 weeks experienced treatment benefit after transitioning to apremilast during Weeks 16–32, with improvements observed as early as Week 20 for modified genital PGA, overall sPGA, BSA, GPI‐NRS and GPSS. DLQI and DLQI‐Q9, which were only measured during the extension phase at Week 32, showed improvements with apremilast treatment at Week 32. Response rates and changes from baseline were maintained or further improved between Weeks 16 and 32 in patients who received apremilast for the full study duration. By the end of the study, 46% of patients in the extension phase showed a modified genital PGA response (48/119 patients in the apremilast/apremilast group and 57/110 patients in the placebo/apremilast group). Baseline characteristics of patients who entered the apremilast extension phase were similar to those for the full intent‐to‐treat population, suggesting no particular trends among those who discontinued treatment before the extension phase.^10^

The safety profile observed across all‐apremilast exposure was consistent with that seen in the placebo‐controlled phase and the expected profile of apremilast.^10^, ^15^ The most common TEAEs were diarrhoea, nausea, headache and nasopharyngitis, and no new safety signals were identified with a longer study duration.^10^, ^15^ TEAEs led to treatment discontinuation in 16 patients (6.3%) throughout the apremilast exposure period.

Post‐hoc analyses by sex showed that both men and women experienced clinical and QoL benefits with apremilast, although greater proportions of women versus men treated with apremilast had treatment responses at Weeks 16 and 32. Additionally, women, who often experience more intense genital psoriasis symptoms and more prevalent sexual dysfunction or distress than men,^1^, ^4^, ^16^ had more substantial mean changes from baseline in DLQI and DLQI‐Q9 at Weeks 16 and 32 when treated with apremilast compared with men.

Novel post‐hoc analyses examined the agreement between clinical responses in modified genital PGA, overall sPGA or GPI‐NRS and QoL improvements in DLQI and DLQI‐Q9 with apremilast treatment at Weeks 16 and 32. In this population of patients with genital psoriasis, the highest agreements in improvement were observed between GPI‐NRS and both DLQI and DLQI‐Q9. These results indicate an agreement between the reduction in genital itch and better general and sexual QoL.

Our results demonstrate the efficacy and safety of apremilast in the treatment of genital psoriasis, a highly burdensome condition that is often neglected. This difficult‐to‐treat disease causes bothersome and embarrassing signs and symptoms such as itching, burning, cracking, scaliness, redness, induration and pain.^4^, ^5^ Due to the perceived stigma surrounding genital psoriasis, patients may be hesitant to discuss it with their physician; therefore, patients may remain underdiagnosed and undertreated.^4^ Furthermore, while the treatment landscape for psoriasis is broad, few treatment options have been specifically studied in genital psoriasis. In the randomized, double‐blind, placebo‐controlled clinical trial IXORA‐Q, subcutaneous interleukin (IL)‐17A antagonist ixekizumab demonstrated significant clearance of genital psoriasis and improvement in genital psoriasis signs and symptoms compared with placebo, with product labelling in the United States revised to include these data.^17^, ^18^, ^19^, ^20^ The biologics secukinumab (an IL‐17A antagonist), guselkumab (an IL‐23 antagonist) and risankizumab (an IL‐23 antagonist) have been evaluated in real‐world observational or open‐label studies.^21^, ^22^, ^23^ There are real‐world data showing the effectiveness of oral deucravacitinib (a tyrosine kinase 2 inhibitor) in moderate to severe psoriasis of special areas, including genital regions.^24^ The DISCREET study fills an important evidence gap, representing the only completed randomized controlled trial in genital psoriasis involving an approved oral therapy.

The DISCREET trial had certain limitations. While placebo‐controlled during the first 16 weeks, there was no placebo control or active comparator during the latter 16 weeks. Sexual function assessment related to psoriasis was limited to DLQI‐Q9. At the same time, the post‐hoc subgroup analysis was performed to compare outcomes by sex; no sex‐specific outcomes (e.g. Sexual Quality of Life Questionnaire for Men, International Index of Erectile Function, Female Sexual Distress Scale, Female Sexual Function Index) were evaluated. Additionally, the majority of patients in this study were male (70.3%), which may have impacted the results of the subgroup analysis. There is some uncertainty about the prevalence of genital psoriasis in men versus women in the literature, with some studies suggesting higher rates in men and another suggesting higher rates in women.^1^, ^2^, ^4^, ^5^ The data could be confounded due to different degrees of comfort among patients in reporting their genital psoriasis. The impact of apremilast on inverse psoriasis was not analyzed in DISCREET. Last, patients were treated for a maximum of 32 weeks on study; studies with longer treatment duration would be beneficial to assess longer‐term impact.

## CONCLUSIONS

Results from the full, 32‐week duration of the phase 3 DISCREET trial concur with the primary results from Week 16, which showed that apremilast significantly reduced clinical findings of genital psoriasis and improved health‐related QoL. Efficacy results at Week 32 were similar across treatment arms; outcomes among patients treated with apremilast throughout the 32‐week study were maintained or improved between Week 16 and Week 32. In DISCREET, outcomes were positive in both sexes treated with apremilast, with greater improvement observed in women. Safety outcomes in patients treated with apremilast at any time during the 32‐week study remained similar to the primary results and were consistent with the known safety profile of apremilast. Based on the consistent clinical efficacy, improvements in symptoms and QoL benefit seen in DISCREET, apremilast appears to be an effective oral therapy in patients with moderate to severe genital psoriasis.

## AUTHOR CONTRIBUTIONS

Study design: SC, MC. Study investigator: JFM, LG, CL, KAP, LCP, PY, AP. Enrolled patients: JFM, LG, CL, KAP, LCP, PY, AP. Data analysis: MC. Data interpretation: All authors. Manuscript preparation: All authors. Manuscript review and revisions: All authors. Final approval of manuscript: All authors.

## FUNDING INFORMATION

This study was sponsored by Amgen Inc.

## CONFLICT OF INTEREST STATEMENT

JFM: AbbVie, Amgen, AstraZeneca, Biogen, Boehringer Ingelheim, Bristol Myers Squibb, Dermavant, Janssen, Lilly, Moonlake, Novartis, Pfizer, Regeneron, Sanofi, Sun Pharma and UCB—consultant and/or investigator. LG: AbbVie, Amgen, Bausch Health, Bristol Myers Squibb, Boehringer Ingelheim, Lilly, Galderma, Janssen, LEO Pharma, Merck Frosst, Novartis, Pfizer, Sun Pharmaceuticals and UCB—consultant, investigator and/or speaker. CL: AbbVie, Actelion, Amgen, Astella, Bausch Health, Boehringer Ingelheim, Dermira, Lilly, Galderma, Janssen, Kyowa Kirin, LEO Pharma, MedImmune, Merck, Novartis, Pfizer, Regeneron, Roche, Sanofi Genzyme, Takeda, UCB and Valeant—principal investigator and/or consultant. KP: AbbVie, Acelyrin, Akros, Alumis, Amgen, Arcutis, Bausch Health/Valeant, Boehringer Ingelheim, Bristol Myers Squibb, Can‐Fite Biopharma, Celltrion, Concert Pharmaceuticals, Dermavant, Dermira, Dice Pharmaceuticals, Dice Therapeutics, Lilly, Evelo Biosciences, Forbion, Galderma, Horizon Therapeutics, Incyte, Janssen, Kymab, Kyowa Kirin, LEO Pharma, Meiji Seika Pharma, Mitsubishi Pharma, Nimbus Therapeutics, Novartis, Pfizer, Reistone, Sanofi‐Aventis/Genzyme, Sandoz, Sun Pharma, Takeda, Tarsus Pharmaceuticals, UCB, and Zai Lab Co.—consultant, investigator and/or speaker. LCP: AbbVie, Alfasigma, Amgen, Amytrx, Bristol Myers Squibb, Lilly, Fibrocell, Galderma, GSK, Kiniksa, Olix, Oneness, Pfizer, Trevi and UCB—investigator. PY: AbbVie, Acelyrin, Akros, Alumis, Amgen, Apollo, Arcutis, Boehringer Ingelheim, Bristol Myers Squibb, Can‐Fite Biopharma, Dermavant, Dice Pharmaceuticals, Dice Therapeutics, Lilly, Galderma, GSK, Horizon Therapeutics, Incyte, Janssen, Novartis, Padagis, Pfizer, Rapt Therapeutics, Regeneron, Sanofi, Sandoz, Sun Pharma, Takeda, and UCB—consultant, investigator and/or speaker. SC: Amgen Inc.—employee and stockholder. HA: Amgen Inc.—employee and stockholder. CD: Amgen Inc.—employee and stockholder. SJ: Amgen Inc.—employee and stockholder at the time of analysis and manuscript development. MC: Amgen Inc.—employee and stockholder. AP: AbbVie, Almirall Hermal, Amgen, Biogen, Boehringer Ingelheim, Bristol Myers Squibb, GSK, Lilly, Galderma, Hexal, Janssen, LEO Pharma, Medac, Merck Serono, Mitsubishi, MSD, Novartis, Pfizer, Tigercat Pharma, Regeneron, Roche, Sandoz Biopharmaceuticals, Schering‐Plough and UCB Pharma—investigator, speaker and/or advisor.

## ETHICAL APPROVAL

This study was approved by an institutional review board or independent ethics committee at each study centre prior to commencement. This study was conducted in compliance with Good Clinical Practice, the International Council for Harmonization Guideline E6, the Declaration of Helsinki and any applicable regulatory requirements.

## ETHICS STATEMENT

Prior to any study‐related procedures, patients provided signed informed consent documentation.

## Supporting information


Data S1.

## Data Availability

Qualified researchers may request data from Amgen clinical studies. Complete details are available at http://www.amgen.com/datasharing.
