# Supplementary material for: Results from the 32‐week, phase 3 DISCREET study of apremilast in patients with moderate to severe genital psoriasis
Source: J Eur Acad Dermatol Venereol. 2025 Dec 10;40(2):274–84. doi: 10.1111/jdv.70110 (PMC12843872; doi:10.1111/jdv.70110)
Supplement: Supplementary file 1 — Data S1. [file JDV-40-274-s001.docx]

# SUPPLEMENTARY MATERIAL

## Methods

Post hoc analyses were conducted at Weeks 16 and 32: efficacy outcomes by sex (categorized by assigned sex at birth); proportion of patients who achieved a modified genital PGA score of 0 (indicating clear genital skin); proportion of patients who achieved a DLQI score of 0 or 1 (indicating unaffected quality of life related to psoriasis); and proportion of patients with a baseline DLQI-Q9 score >0 who achieved a DLQI-Q9 score of 0 (indicating no sexual difficulty related to psoriasis).

A post hoc analysis was conducted to assess the agreement between improvements in clinical outcomes and symptoms (i.e., a modified genital PGA score or overall sPGA score of 0, or a GPI-NRS response at Week 16 or Week 32) and improvements in QoL outcomes (ie, DLQI response, defined as achieving a score of 0 or 1 at Week 16 or Week 32, or DLQI-Q9 response, defined as achieving a score of 0 or 1 at Week 16 or Week 32) with apremilast treatment. This analysis was conducted for patients randomized to apremilast at baseline. The agreement between clinical and QoL improvements with apremilast treatment was calculated by dividing the number of patients who responded to both endpoints by the total number of patients with available data for both endpoints measured at that timepoint.

## Table S1. Baseline demographics and clinical characteristics by sex of patients in the intent-to-treat population

|  | **Placebo** | | | **Apremilast** | | | | **Total** | | |  |
| --- | --- | --- | --- | --- | --- | --- | --- | --- | --- | --- | --- |
|  | **Men n=102** | **Women n=44** | | **Men n=100** | **Women n=43** | | | **Men n=202** | **Women n=87** | |  |
| Age, y |  |  | |  | |  | |  | |  |  |
| Mean (SD) | 47.4 (15.0) | 44.2 (12.8) | | 42.5 (11.7) | | 46.0 (16.4) | | 45.0 (13.7) | | 45.1 (14.6) | |
| Median (IQR) | 46.5 (36.0, 57.0) | 44.5 (33.5, 54.0) | | 41.5 (34.0, 51.0) | | 48.0 (30.0, 59.0) | | 44.0 (35.0, 55.0) | | 46.0 (33.0, 58.0) | |
| Weight, kg |  |  | |  | |  | |  | |  |  |
| Mean (SD) | 93.8 (21.6) | 76.8 (16.2) | | 93.3 (18.1) | | 85.1 (26.0) | | 93.5 (19.9) | | 80.9 (21.9) | |
| Median (IQR) | 91.0 (79.4, 104.0) | 73.1 (63.8, 88.8) | | 88.7 (83.0, 101.6) | | 80.7 (63.6, 104.8) | | 89.8 (81.7, 102.0) | | 77.0 (63.6, 95.7) | |
| Body mass index, kg/m^2^ |  |  | |  | |  | |  | |  |  |
| Mean (SD) | 30.1 (6.4) | 29.4 (6.2) | | 29.4 (5.3) | | 31.9 (8.8) | | 29.8 (5.9) | | 30.6 (7.7) |  |
| Median (IQR) | 29.1 (25.5, 33.4) | 28.7 (24.4, 35.2) | | 28.7 (26.2, 31.3) | | 30.9 (25.5, 38.3) | | 28.9 (25.9, 32.6) | | 28.9 (24.7, 37.4) |  |
| Duration of psoriasis, y |  |  | |  | |  | |  | |  |  |
| Mean (SD) | 15.9 (13.2) | 13.0 (12.3) | 14.1 (11.7) | | 16.8 (15.0) | | 15.0 (12.5) | | | 14.9 (13.8) | |
| Median (IQR) | 12.5 (5.0, 23.5) | 9.1 (3.1, 20.5) | 10.9 (4.1, 20.2) | | 14.3 (4.0, 28.2) | | 11.8 (4.7, 20.7) | | | 10.0 (3.4, 23.1) | |
| Duration of genital psoriasis, y |  |  | |  | |  | |  | |  |  |
| Mean (SD) | 13.3 (13.2) | 8.7 (9.9) | | 10.8 (10.5) | | 11.5 (12.0) | | 12.1 (12.0) | | 10.1 (11.0) | |
| Median (IQR) | 8.3 (3.2, 19.1) | 4.3 (1.8, 12.4) | | 7.6 (2.8, 15.1) | | 5.4 (2.0, 19.3) | | 7.7 (2.9, 17.8) | | 4.9 (1.8, 15.3) | |
| Modified genital PGA score, n (%) |  |  | |  | |  | |  | |  |  |
| 3 (moderate) | 86 (84.3) | 42 (95.5) | | 86 (86.0) | | 37 (86.0) | | 172 (85.1) | | 79 (90.8) |  |
| 4 (severe) | 16 (15.7) | 2 (4.5) | | 14 (14.0) | | 6 (14.0) | | 30 (14.9) | | 8 (9.2) |  |
| Overall sPGA score, n (%) |  |  | |  | |  | |  | |  |  |
| 3 (moderate) | 89 (87.3) | 41 (93.2) | | 88 (88.0) | | 38 (88.4) | | 177 (87.6) | | 79 (90.8) |  |
| 4 (severe) | 12 (11.8) | 3 (6.8) | | 12 (12.0) | | 5 (11.6) | | 24 (11.9) | | 8 (9.2) |  |
| BSA, % |  |  | |  | |  | |  | |  |  |
| Mean (SD) | 8.9 (4.9) | 7.7 (5.0) | | 10.9 (12.7) | | 10.4 (14.0) | | 9.9 (9.6) | | 9.0 (10.5) | |
| Median (IQR) | 8.0 (5.0, 12.0) | 6.5 (3.0, 10.5) | | 8.5 (4.0, 12.0) | | 6.5 (5.0, 12.0) | | 8.0 (4.0, 12.0) | | 6.5 (4.0, 11.0) | |
| <10%, n (%) | 56 (54.9) | 28 (63.6) | | 54 (54.0) | | 28 (65.1) | | 110 (54.5) | | 56 (64.4) |  |
| ≥10%, n (%) | 46 (45.1) | 16 (36.4) | | 46 (46.0) | | 15 (34.9) | | 92 (45.5) | | 31 (35.6) |  |
| DLQI |  |  | |  | |  | |  | |  |  |
| Mean (SD) | 12.0 (6.9) | 14.5 (6.6) | | 12.6 (7.2) | | 14.8 (6.6) | | 12.3 (7.0) | | 14.7 (6.6) |  |
| Median (IQR) | 10.0 (7.0, 17.0) | 13.0 (10.0, 19.0) | | 12.0 (7.0, 18.0) | | 14.0 (11.0, 19.0) | | 11.0 (7.0, 18.0) | | 13.5 (10.0, 19.0) |  |
| DLQI-Q9 |  |  | |  | |  | |  | |  |  |
| Mean (SD) | 1.3 (1.0) | 1.6 (1.1) | | 1.3 (1.1) | | 1.5 (1.2) | | 1.3 (1.1) | | 1.5 (1.2) |  |
| Median (IQR) | 1.0 (0.0, 2.0) | 2.0 (1.0, 3.0) | | 1.0 (0.0, 2.0) | | 1.5 (0.0, 3.0) | | 1.0 (0.0, 2.0) | | 2.0 (0.0, 3.0) |  |
| GPI-NRS |  |  | |  | |  | |  | |  |  |
| Mean (SD) | 5.9 (2.5) | 7.6 (1.8) | | 6.2 (2.3) | | 7.6 (1.8) | | 6.1 (2.4) | | 7.6 (1.8) |  |
| Median (IQR) | 6.0 (4.0, 8.0) | 8.0 (7.0, 9.0) | | 7.0 (5.0, 8.0) | | 8.0 (7.0, 9.0) | | 7.0 (5.0, 8.0) | | 8.0 (7.0, 9.0) |  |

The n values reflect the number of patients who entered the apremilast extension phase; the actual number of patients available for each parameter may vary.
BSA, body surface area; DLQI, Dermatology Life Quality Index; DLQI-Q9, Dermatology Life Quality Index Question 9; GPI-NRS, Genital Psoriasis Itch Numeric Rating Scale; Genital PGA, static Physician’s Global Assessment of Genitalia; IQR, interquartile; SD, standard deviation; sPGA, static Physician’s Global Assessment.

## Table S2. Agreement between improved clinical and QoL outcomes among patients randomized to apremilast

| Consistent improvement, n/N (%) | DLQI = 0/1^a^ | | DLQI-Q9 = 0/1^b^ | |
| --- | --- | --- | --- | --- |
|  | Week 16 | Week 32 | Week 16 | Week 32 |
| Modified genital PGA = 0^c^ | 10/118 (8.5) | 10/100 (10.0) | 27/118 (22.9) | 27/100 (27.0) |
| Overall sPGA = 0^d^ | 3/118 (2.5) | 5/100 (5.0) | 7/118 (5.9) | 13/100 (13.0) |
| GPI-NRS response^e^ | 13/101 (12.9) | 17/84 (20.2) | 43/101 (42.6) | 44/84 (52.4) |

## Values were calculated based on nonmissing data of the pair of clinical and QoL parameters at each time point for patients initially randomized to apremilast at baseline. Percentages represent the proportion of these patients who achieved responses to both specified clinical and QoL parameters. ^a^DLQI total score ranges from 0 to 30, with higher scores corresponding with poorer quality of life; a score of 0 or 1 indicates that psoriasis had no effect on quality of life. ^b^DLQI-Q9 asks, “Over the last week, how much has your skin caused any sexual difficulties?”, with scores ranging from 0 to 3; a score of 0 or 1 indicates little to no impact. ^c^Modified genital PGA score of 0 indicates clear genital skin. ^d^sPGA score of 0 indicates clear skin. ^e^GPI-NRS score ranges from 0 (no itch) to 10 (worst itch imaginable); response is defined as a ≥4-point reduction in score from baseline in patients with a baseline score ≥4.

## DLQI, Dermatology Life Quality Index; DLQI-Q9, Dermatology Life Quality Index Question 9; Genital PGA, static Physician’s Global Assessment of Genitalia; GPI-NRS, Genital Psoriasis Itch Numeric Rating Scale; QoL, Quality of Life; sPGA, static Physician’s Global Assessment.

## Figure S1.

Proportion of patients achieving modified genital PGA clearance (score of 0) at Weeks 16 and 32 (**a**), proportion of patients achieving DLQI score of 0 or 1 at Weeks 16 and 32 (**b**), or a proportion of patients with a baseline DLQI-Q9 score >0 achieving DLQI-Q9 score of 0 (**c**).

(a)


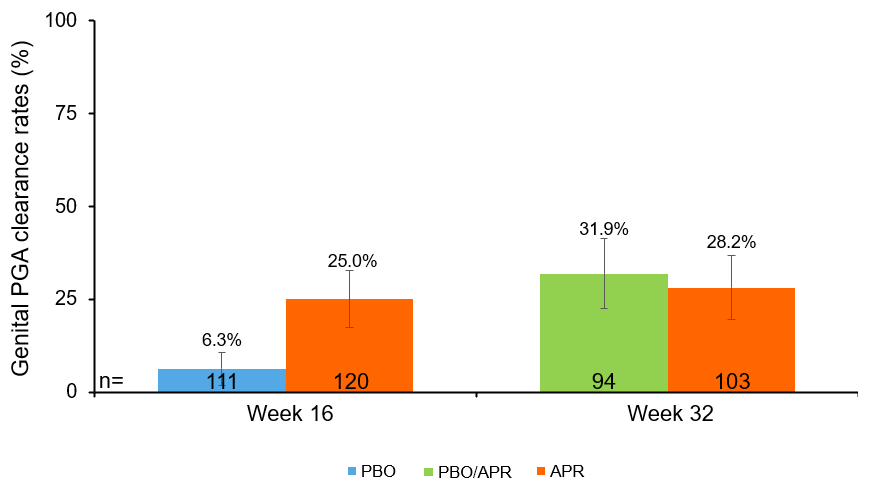


## (b)
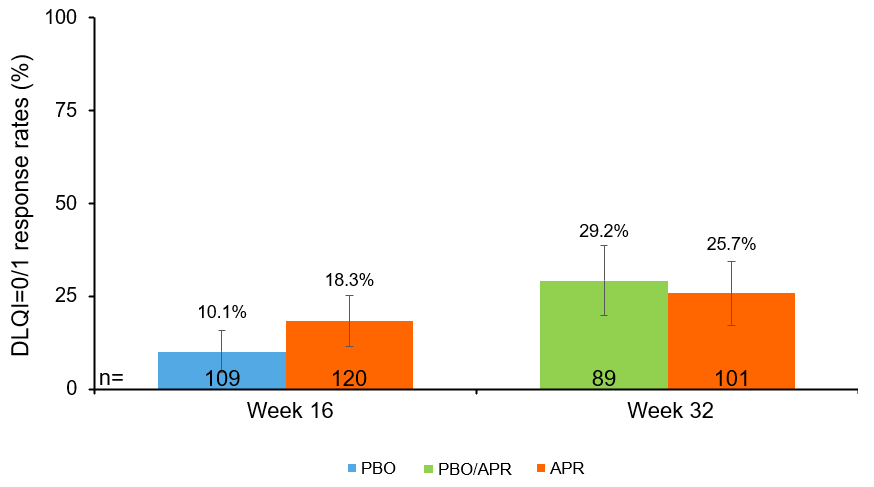


(c)


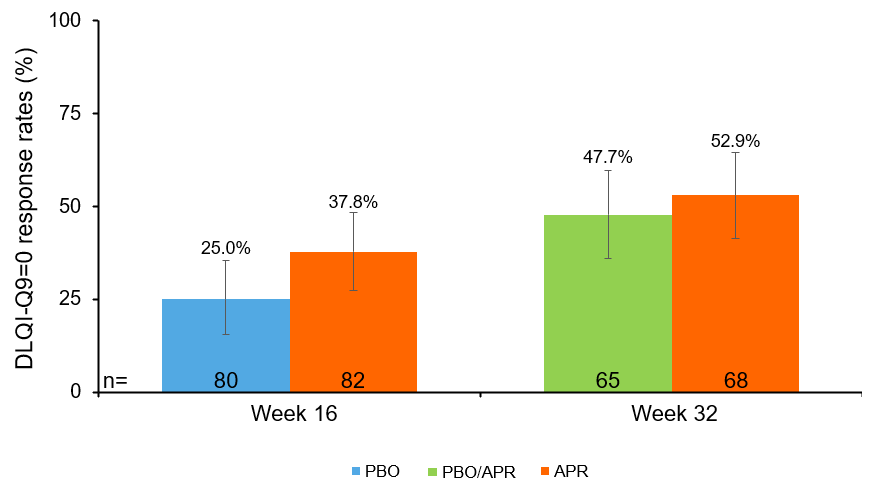


Results are based on data as observed at each of the individual time points. Error bars represent 95% CI.

## APR, apremilast; CI, confidence interval; DLQI, Dermatology Life Quality Index; DLQI-Q9, Dermatology Life Quality Index Question 9; PBO, placebo; PGA, Physician’s Global Assessment.

## Figure S2. Outcomes by sex at Weeks 16 and 32, including genital PGA responses (a), overall sPGA responses (b), GPI-NRS responses (c), change from baseline in DLQI (d), and change from baseline in DLQI-Q9 (e). Week 32 includes all patients who received apremilast in the extension phase whether initiated from randomization or switched from placebo at Week 16. In panels (a–c), missing data were imputed using the multiple imputation method for Week 16 response rates, while nonresponder imputation was used for Week 32 response rates. Panel (c) includes patients with a baseline GPI-NRS score of ≥4. Data shown in panels (d–e) are least-squares means based on the mixed effects model for repeated measures during the PBO-controlled phase (Weeks 0–16) and based on data as observed during the apremilast extension phase (Weeks 16–32). Error bars represent 95% CI. APR, apremilast 30 mg twice daily; DLQI, Dermatology Life Quality Index; DLQI-Q9, Dermatology Life Quality Index Question 9; genital PGA, static Physician’s Global Assessment of Genitalia; GPI-NRS, Genital Psoriasis Itch Numeric Rating Scale; PBO, placebo; sPGA, static Physician’s Global Assessment.

(a)


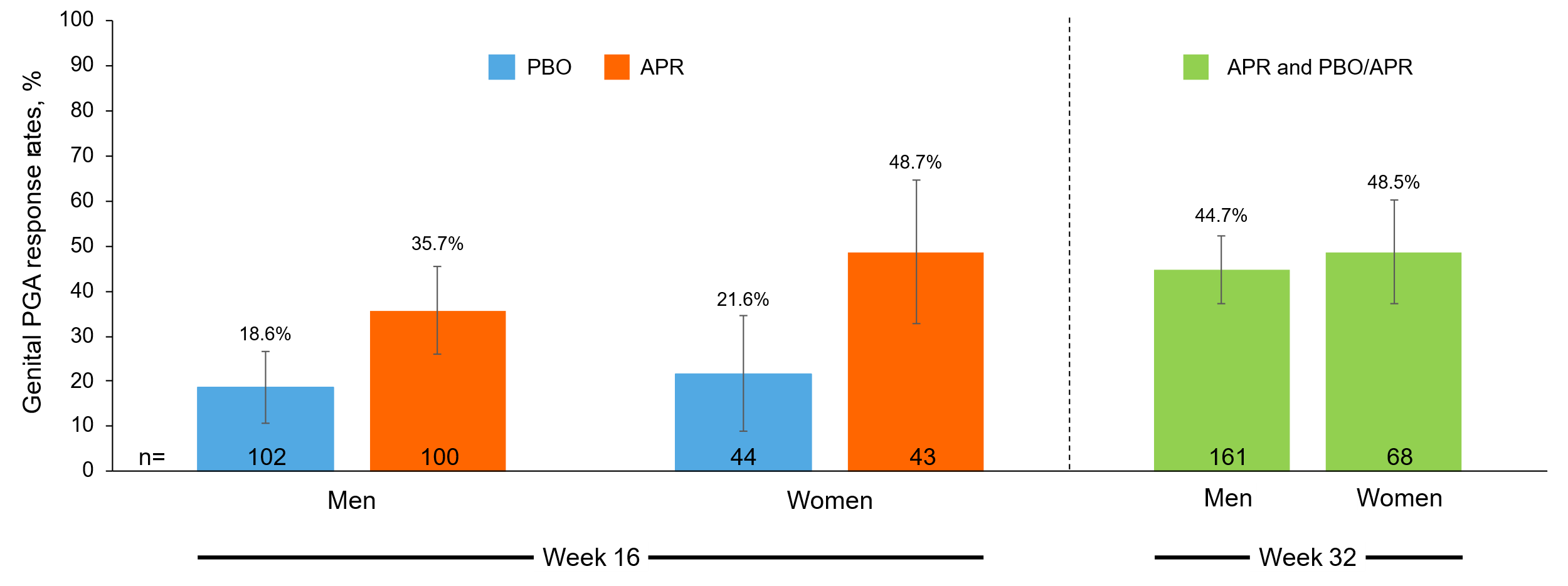


(b)


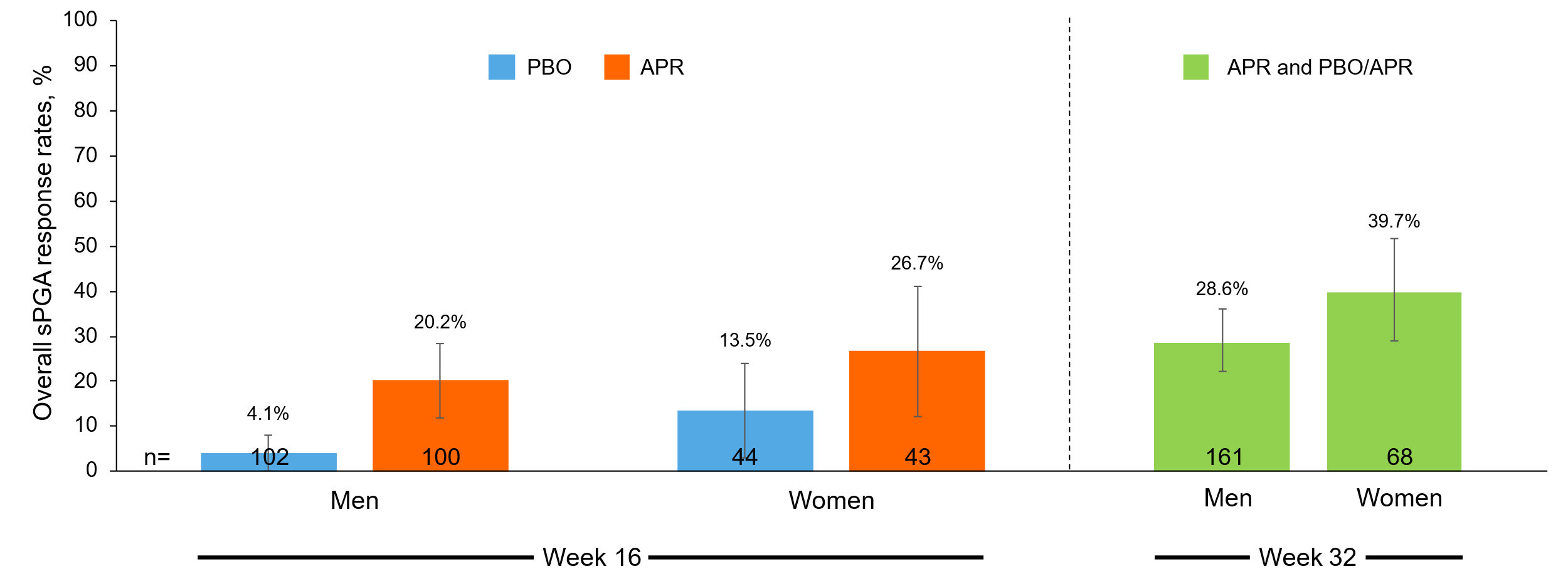


(c)


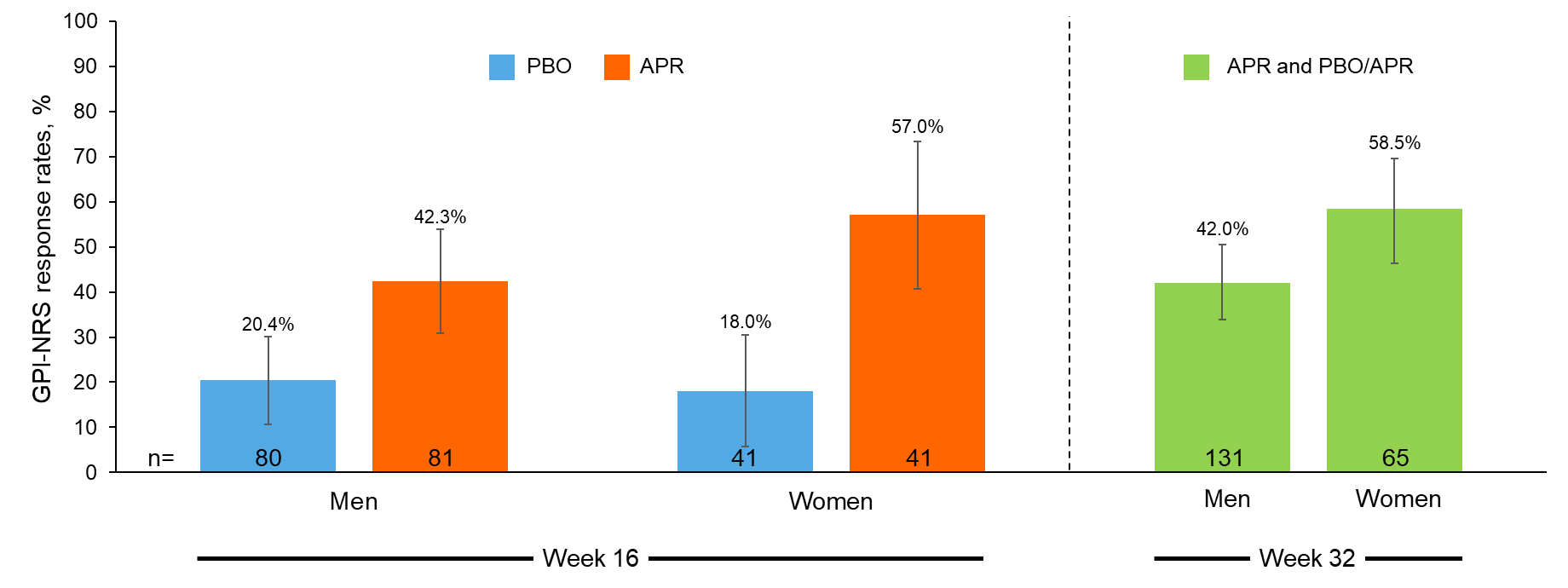


(d)


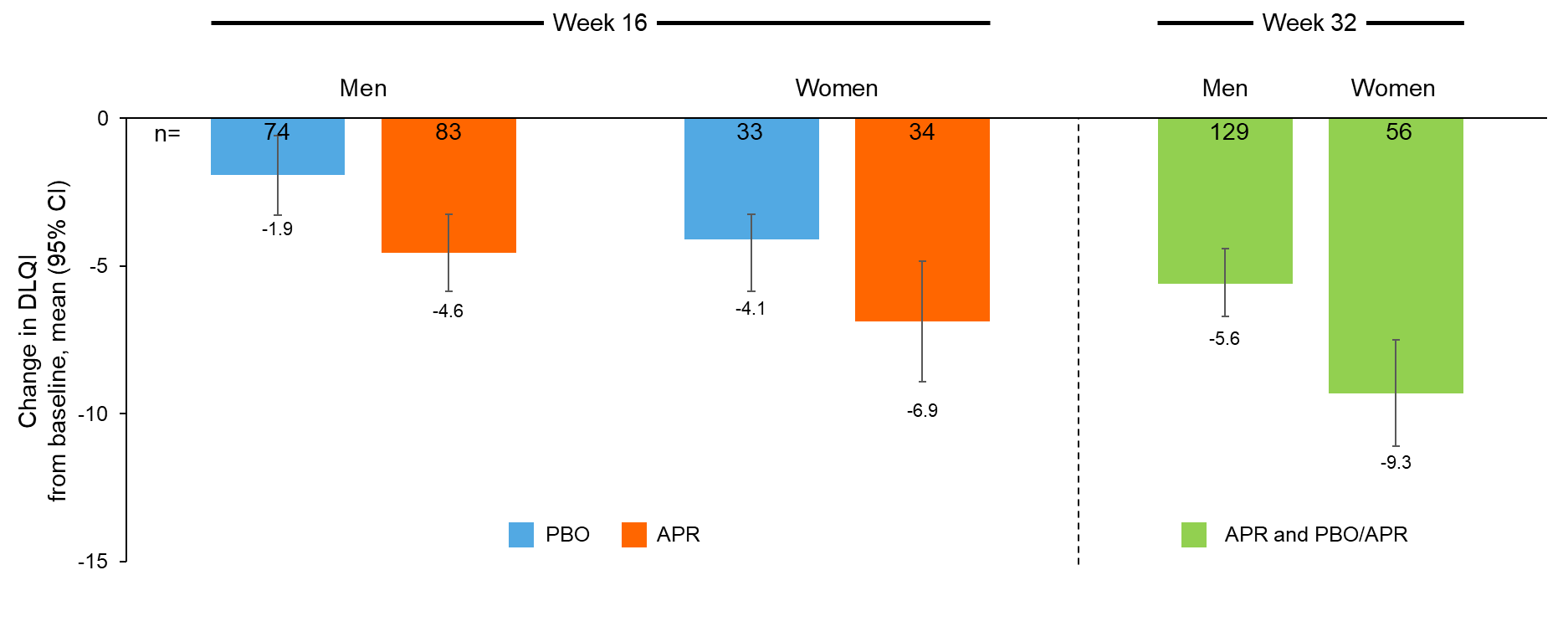


(e)

**
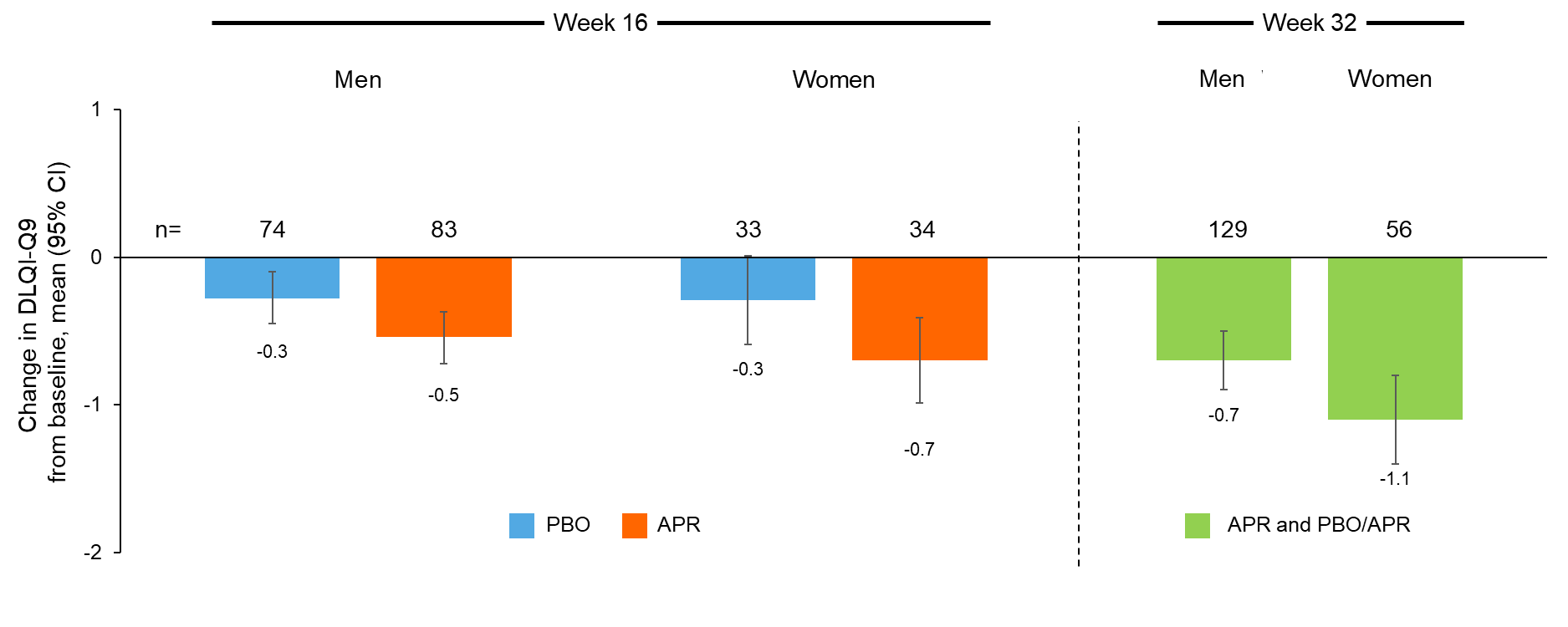
**
